# Regional covariance of white matter hyperintensity volume patterns associated with hippocampal volume in healthy aging

**DOI:** 10.3389/fnagi.2024.1349449

**Published:** 2024-03-08

**Authors:** Emily J. Van Etten, Pradyumna K. Bharadwaj, Matthew D. Grilli, David A. Raichlen, Georg A. Hishaw, Matthew J. Huentelman, Theodore P. Trouard, Gene E. Alexander

**Affiliations:** ^1^Department of Psychology, University of Arizona, Tucson, AZ, United States; ^2^Evelyn F. McKnight Brain Institute, University of Arizona, Tucson, AZ, United States; ^3^Department of Neurology, University of Arizona, Tucson, AZ, United States; ^4^Human and Evolutionary Biology Section, Department of Biological Sciences, University of Southern California, Los Angeles, CA, United States; ^5^Department of Anthropology, University of Southern California, Los Angeles, CA, United States; ^6^Neurogenomics Division, The Translational Genomics Research Institute (TGen), Phoenix, AZ, United States; ^7^Arizona Alzheimer’s Consortium, Phoenix, AZ, United States; ^8^Department of Biomedical Engineering, University of Arizona, Tucson, AZ, United States; ^9^Department of Psychiatry, University of Arizona, Tucson, AZ, United States; ^10^Neuroscience Graduate Interdisciplinary Program, University of Arizona, Tucson, AZ, United States; ^11^Physiological Sciences Graduate Interdisciplinary Program, University of Arizona, Tucson, AZ, United States

**Keywords:** regional white matter hyperintensity volume, hippocampal volume, brain aging, subjective memory complaints, scaled subprofile model, multivariate analyses

## Abstract

Hippocampal volume is particularly sensitive to the accumulation of total brain white matter hyperintensity volume (WMH) in aging, but how the regional distribution of WMH volume differentially impacts the hippocampus has been less studied. In a cohort of 194 healthy older adults ages 50–89, we used a multivariate statistical method, the Scaled Subprofile Model (SSM), to (1) identify patterns of regional WMH differences related to left and right hippocampal volumes, (2) examine associations between the multimodal neuroimaging covariance patterns and demographic characteristics, and (3) investigate the relation of the patterns to subjective and objective memory in healthy aging. We established network covariance patterns of regional WMH volume differences associated with greater left and right hippocampal volumes, which were characterized by reductions in left temporal and right parietal WMH volumes and relative increases in bilateral occipital WMH volumes. Additionally, we observed lower expression of these hippocampal-related regional WMH patterns were significantly associated with increasing age and greater subjective memory complaints, but not objective memory performance in this healthy older adult cohort. Our findings indicate that, in cognitively healthy older adults, left and right hippocampal volume reductions were associated with differences in the regional distribution of WMH volumes, which were exacerbated by advancing age and related to greater subjective memory complaints. Multivariate network analyses, like SSM, may help elucidate important early effects of regional WMH volume on brain and cognitive aging in healthy older adults.

## 1 Introduction

With the older adult population rising at a rapid rate, further research is needed to better understand how different factors may influence brain and cognitive aging. Vascular risk factors are highly prevalent in older adults (Mozaffarian et al., 2015) and may amplify age-related brain atrophy and cognitive decline (Debette et al., 2011). One vascular health-related factor often investigated in aging research includes white matter hyperintensity (WMH) volume, a neuroimaging marker of white matter lesion load related to cerebral small vessel disease (Prins and Scheltens, 2015). Previous findings have observed that elevated total WMH volumes are associated with widespread decreases in gray matter, particularly in frontal and temporoparietal regions (Kern et al., 2017), and poorer cognitive functions (Gunning-Dixon and Raz, 2000; Moura et al., 2019) in older adults. Further, hippocampal volume atrophy, an important neuroanatomical marker associated with objective and subjective memory (Head et al., 2008; Striepens et al., 2010) and a hallmark feature of Alzheimer’s disease (AD; Jack et al., 2000), appears to be especially vulnerable to accumulation of total WMH volume in aging (Fiford et al., 2017).

Compared to total WMH volume, the impact of specific lobar WMH volumes on the hippocampus has been less studied, but the regional distribution of WMH’s may have differential effects on brain aging, including hippocampal volume (Van Etten et al., 2021). Moreover, the limited studies have been variable, with separate studies observing significant associations between different regional lobar WMH volumes (i.e., frontal, parietal, temporal, or occipital) and hippocampal volume in older adults with and/or without cognitive impairment (Guzman et al., 2013; Rizvi et al., 2018; Vipin et al., 2018; Van Etten et al., 2021). All previous studies, however, have applied univariate analysis techniques that do not consider patterns of covariance across regional WMH volumes. Further research with multivariate network analyses that may be more sensitive in identifying patterns of regional differences (Alexander and Moeller, 1994) could help elucidate how the regional distribution of WMH volume throughout the brain impact hippocampal volume in healthy aging.

Reductions in brain structure as a result of increases in WMH volume may, in turn, impact cognitive performance, including memory decline (Rizvi et al., 2018). Rizvi et al. (2018) found medial temporal thickness and volume mediated the relation between regional WMH volume and memory, suggesting regional WMH volume may be associated with poorer memory performance through its impact on medial temporal lobe structures, such as the hippocampus. Additionally, regional WMH volume may influence subjective memory complaints, which are self-reported, perceived declines in memory abilities (Morrison et al., 2023). Subjective memory complaints may be an important early indicator of cognitive aging and risk for AD (Reisberg and Gauthier, 2008), representing potential memory changes not yet detected on objective memory measures (Reisberg et al., 2010; Jessen et al., 2014). A recent study found increased subjective memory complaints were associated with greater temporal and parietal WMH volume burden, indicating regional WMH volume differences, particularly in temporal and parietal lobes, may be sensitive to subjective cognitive decline (Morrison et al., 2023).

In this study, we used a multivariate analysis method, the Scaled Subprofile Model (SSM; Moeller et al., 1987), to derive network covariance patterns that reflect the relative differences in regional WMH volumes associated with left and right hippocampal volumes in a cohort of cognitively healthy older adults. This method has been used in many human and non-human animal structural neuroimaging studies (Alexander et al., 2006, 2008, 2012a, 2020; Brickman et al., 2007, 2008; Bergfield et al., 2010; Kern et al., 2017), but has not yet been applied to studies of regional WMH volume. In a cohort of healthy older adults 50–89 years of age, we sought to (1) identify patterns of regional WMH differences related to left and right hippocampal volumes, (2) examine associations of the multimodal neuroimaging covariance patterns with demographic (age, sex, education) and other important health risk factors [apolipoprotein E (APOE) ε4 status and vascular risk], and (3) investigate whether the hippocampal-related regional WMH patterns were related to subjective memory complaints and objective memory performance in healthy aging.

## 2 Method

### 2.1 Participants

Participants included 194 healthy older adults ages 50–89 years who were drawn from a cohort of 210 community-dwelling adults. Before enrollment in the full cohort, participants were given a comprehensive medical screen and a neurologist performed a physical and neurological examination. Participants were excluded for history of major neurological, medical, or psychiatric illnesses. Additionally, those who had a Mini Mental Status Exam (MMSE; Folstein et al., 1975) score less than 26 or a Hamilton Depression Rating Scale (HAM-D; Hamilton, 1960) score greater than 9 were excluded from the study. Participants provided written consent and all procedures were approved by the Institutional Review Board and the University of Arizona.

For this study, after log transformation, regional WMH volume outliers that were ± 3.5 or more standard deviations (SDs) away from the mean in one or more hemispheric lobes were removed (*n* = 16). The resulting sample (*n* = 194), was predominately White (94.8%), had an average age of 70.87 years (SD = 10.08) and education of 15.91 years (SD = 2.59; see Table 1).

**Table 1:** Participant demographic, cognitive, and neuroimaging characteristics.

Variable	
*N*	194
Age [years; *M* (*SD*)]	70.87 (10.07)
Education [years; *M* (*SD*)]	15.91 (2.59)
Sex (F/M)	93/101
MMSE [*M* (*SD*)]	28.92 (1.24)
APOE ε4 Status^a^ (Y/N)	57/134
GDS [*M* (*SD*)]	0.97 (1.64)
MFQ [*M* (*SD*)]	5.06 (1.16)
SRT Sum Recall^b^ [*M* (*SD*)]	102.77 (20.57)
SRT CLTR^b^ [*M* (*SD*)]	60.97 (37.40)
Right Hippocampal Volume^c^ [*M* (*SD*)]	4067.30 (560.34)
Left Hippocampal Volume^c^ [*M* (*SD*)]	4193.05 (561.25)

Means (standard deviations) for our sample. ^a^*n* = 191, ^b^*n* = 190, ^c^cm^3^. F/M, Female/Male; Y/N, Yes/No; MMSE, Mini Mental Status Exam; GDS, Geriatric Depression Scale; MFQ, Memory Functioning Questionnaire; SRT, Selective Reminding Test; CLTR, Consistent Long-Term Retrieval.

### 2.2 APOE genotyping

As detailed in our previous studies (Van Etten et al., 2021; Song et al., 2023), APOE genotype was established with extracted DNA assayed via restriction fragment length polymorphism according to published methods (Addya et al., 1997).

### 2.3 Vascular risk

A vascular risk score was created based on the presence of hypertension, cholesterol, history of smoking, and overweight body mass index (BMI), which are well-established vascular risk factors for dementia and AD (Kivipelto et al., 2005). The presence or absence of a factor was determined by self-report for hypertension, cholesterol, and history of smoking, and each of these were assigned a value of 0 if absent and a value of 1 if present. BMI was computed with measured height and weight and individuals with a BMI below 25 were assigned a value of 0 and those with a BMI equal to or greater than 25 (overweight or higher) were assigned a value of 1. A vascular risk score was calculated as a sum of the factors.

### 2.4 Subjective memory complaints

Subjective memory complaints were measured using a portion of the Memory Functioning Questionnaire, which is a reliable measure for evaluating subjective memory complaints (Gilewski et al., 1990). We focused on the question asking participants to rate their overall memory problems on a 1–7 scale, with lower scores indicating greater memory complaints, as we have previously found this question to be sensitive to differences in hippocampal volume (Van Etten et al., 2020).

### 2.5 Objective memory measures

Participants were administered the 12-item, 12-trial version of the Selective Reminding Test (SRT; Buschke, 1973) as part of a larger neuropsychological battery. The SRT is a verbal list-learning task in which participants were asked to remember a list of 12 unrelated words and recalled the words after each trial. For each subsequent trial, participants were selectively reminded of the words they did not recall on the previous trial. After a 30-min delay, participants were asked to recall all words again. For the present study, we used total sum recall (number of words recalled across immediate trials) and consistent long-term retrieval (CLTR; words consistently recalled across at least three successive trials without interruption), measures sensitive to cognitive aging (Alexander et al., 2012b).

### 2.6 Image acquisition

Volumetric T1-weighted 3D Spoiled Gradient Echo (SPGR; slice thickness = 1.0 mm, TR = 5.3 ms, TE = 2.0 ms, TI = 500 ms, FA = 15, matrix = 256 × 256, FOV = 25.6 cm) and T2 Fluid-Attenuation Inversion Recovery (FLAIR) magnetic resonance imaging (MRI) scans (slice thickness = 2.6 mm, TR = 11,000 ms, TE = 120 ms, TI = 2,250 ms, flip angle = 90, matrix = 256 × 256, FOV = 25.0 cm) were acquired on a 3 T GE Signa Excite scanner (General Electric, Milwaukee, WI).

### 2.7 Image processing

#### 2.7.1 Hippocampal volumes

T1-weighted 3D volumetric MRIs were processed using FreeSurfer v5.3, and the technical procedures involved have been previously described in detail (Fischl et al., 2004). The subcortical segmentation stream was used to acquire right and left hippocampal volumes, and each were visually inspected and then re-processed as needed. Using T1 scans, total intracranial volume (TIV) for each participant was determined in native brain space (Alexander et al., 2012a) with Statistical Parametric Mapping (SPM12; Wellcome Trust Centre for Neuroimaging, London, UK).

#### 2.7.2 Regional WMH volumes

Our approach for processing regional WMH volumes has been detailed in prior publications (Franchetti et al., 2020; Van Etten et al., 2021). T1 and T2 FLAIR scans were used to compute total WMH volume with the lesion segmentation toolbox (LST; Schmidt et al., 2012) for SPM12, using the multispectral lesion growth algorithm (LGA) approach. Manually segmented reference WMH maps were produced and reviewed to consensus by expert raters with ITK-SNAP^1^ (Yushkevich et al., 2006) to compare to the LGA accuracy across an array of kappa thresholds (0.05–1.00) in a subset of 35 participants. The overlap between the LGA-generated global WMH volume maps from each kappa level and the manually segmented reference global WMH maps were compared with the dice coefficient, which was highest at the kappa threshold of 0.35. The LGA lesion probability maps at the optimal kappa threshold for our sample (0.35) were then generated for all participants and visually inspected for accuracy to estimate total WMH volumes.

Briefly, to determine regional WMH volumes (Franchetti et al., 2020; Van Etten et al., 2021), FreeSurfer v5.3 was used to process the MNI152 template. The regional labels from the Desikan-Killiany atlas (Desikan et al., 2006) were combined according to FreeSurfer’s standard lobar schema and propagated into the white matter, to produce labels for the left and right hemispheres of the four major lobes and generate the lobar template. Next, the FreeSurfer MNI152 lobar template was non-linearly registered to each participant’s T1 scan using the Advanced Normalization Tools’ Greedy SyN algorithm (ANTs; Avants et al., 2011), to create native space lobar ROIs. These T1 native space lobar ROIs were used to extract the regional WMH volumes for each participant with the LST-generated lesion probability maps. After log transformation of the regional WMH volumes, outliers (*n* = 16) that were ± 3.5 SD away from the mean for any hemispheric lobe were excluded, leaving 194 participants with regional WMH values for inclusion in the network covariance analyses.

### 2.8 Statistical analyses

#### 2.8.1 Network covariance patterns

Two SSM analyses were performed using MATLAB (Math Works, Natick, Massachusetts, USA; Moeller et al., 1987; Habeck et al., 2005) to determine the regional network patterns of WMH differences associated with right and left hippocampal volume, separately. Region-wise and subject-wise means of natural log transformed lobar WMH values were subtracted from each cell. A modified principal component analysis was then performed on the residual natural log-transformed regional WMH volume data. This produced a set of components of the region by subject data, reflecting regional covariance patterns and corresponding individual scores that represent the degree to which each participant expresses the patterns (Alexander and Moeller, 1994). Multiple linear regressions with the Akaike Information Criteria (AIC) were used to determine point estimates from the linear combination of SSM components that best predicted right or left hippocampal volume (Burnham and Anderson, 2004). A bootstrap resampling procedure with 10,000 iterations was performed on the point estimates from the SSM to provide confidence intervals for the observed regional pattern weights (Efron and Tibshirani, 1994; Habeck et al., 2005). Follow-up Pearson correlations were performed to determine which regional WMH volumes were significantly, univariately associated with left and right hippocampal volumes. Additionally, we conducted follow-up SSM analyses with hippocampal volume averaged across hemispheres and after statistically removing the average hippocampal volume from the left and right hippocampal values to further test for lateralized hemispheric pattern differences (see Supplementary material).

#### 2.8.2 Regressions

Linear regressions were used to examine relationships between the left or right hippocampal-related regional WMH patterns and important demographic and participant health characteristics. Two regressions were conducted, with each including age, sex, education, TIV, APOE ε4 status, and vascular risk score as independent variables and the covariance patterns as separate dependent variables. Since 3 individuals were missing APOE ε4 status data, these follow-up analyses consisted of 191 participants.

Linear regressions were also performed with the left and right hippocampal-related regional WMH patterns as the independent variables with objective memory performance and subjective memory complaints as the dependent variables. Age, sex, education, TIV, APOE ε4 status, and vascular risk score were included as covariates. The analyses with memory performance included 190 individuals, as 1 individual was missing memory data.

#### 2.8.3 Exploratory analyses

We conducted exploratory analyses with linear regressions to examine whether demographic and clinical characteristics (including age, sex, education, APOE ε4 status, and vascular risk score) moderated the relationships of each hippocampal-related regional WMH patterns with objective memory performance and subjective memory complaints as the dependent variables (see Supplementary material).

## 3 Results

### 3.1 Network covariance patterns

**Figure 1:**
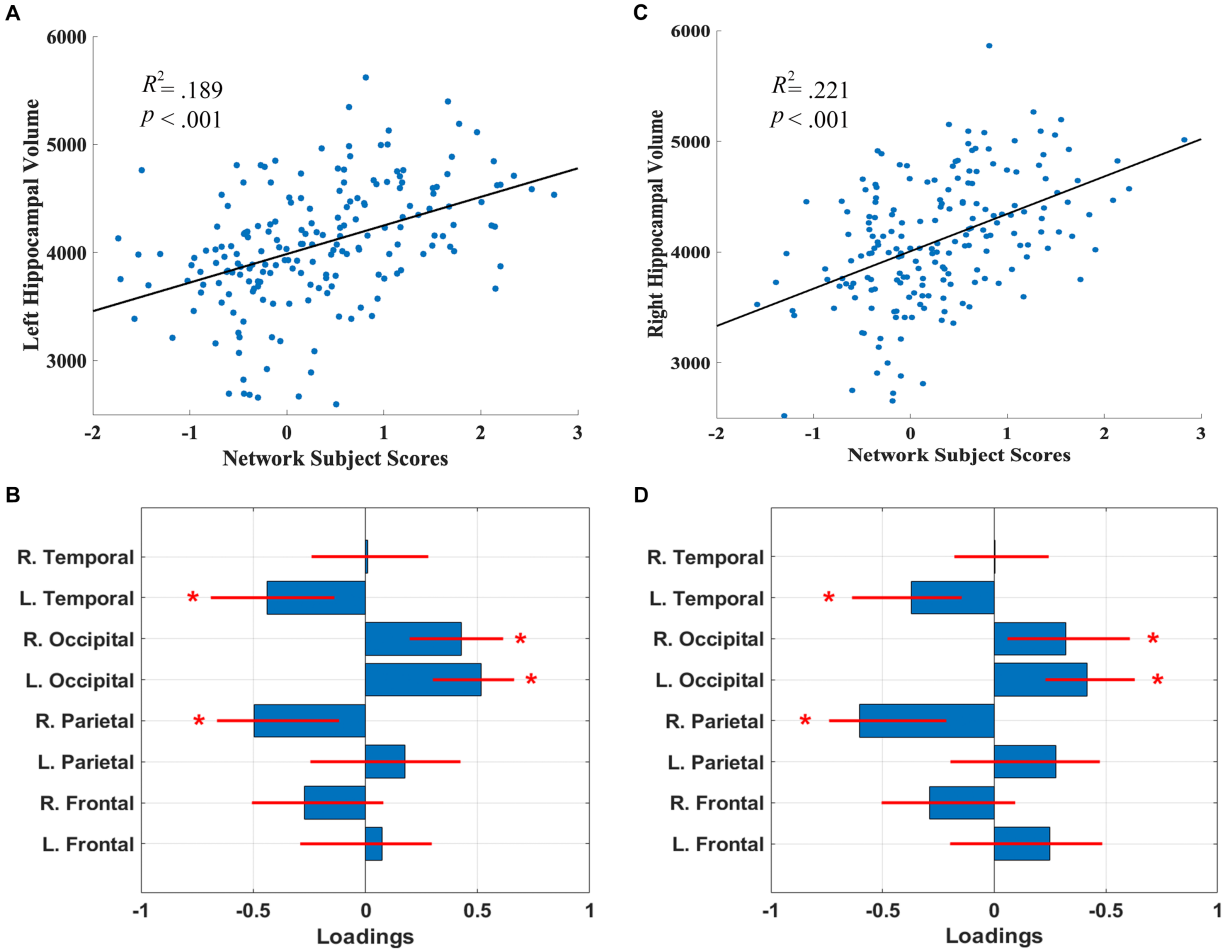
Left **(A)** and right **(C)** hippocampal network subject scores and hippocampal volume. The subject scores of the left and right hippocampal-related network patterns were each derived from the first 5 components. The scatterplots show that greater hippocampal volumes were associated with higher expression of the network patterns. Left **(B)** and right **(D)** hippocampal-related regional WMH loadings for the SSM network pattern of regional WMH volumes. Blue bars indicate point estimates for the loadings and red lines indicate the 95% confidence intervals. Asterisks reflect significant ROIs contributing to the covariance pattern.

The SSM analyses, with the AIC method, identified the linear combination of components that best predicted left and right hippocampal volume. For the left hippocampal-related regional WMH pattern, the model included the first 5 components, and accounted for 18.9% of the variance in left hippocampal volume (*β* = 264.31, *p* = 2.51E-8) with higher expression of the network pattern related to greater volume. Bootstrap re-sampling of the linearly combined pattern of the first 5 components was characterized by reductions of left temporal and right parietal WMH volumes and relative increases in left and right occipital WMH volumes (see Figures 1A,B). After controlling for age and TIV, the pattern significantly predicted left hippocampal volume (*β* = 81.86, *p* = 0.030). The pattern also significantly predicted left hippocampal volume (*β* = 215.10, *p* = 9.06E-7), after we controlled for total WMH volume.

For the right hippocampal-related regional WMH pattern, the model included the first 5 components, and accounted for 21.2% of the variance in right hippocampal volume (*β* = 337.82, *p* = 1.37E-9) with higher expression of the network pattern associated with greater volume. Bootstrap re-sampling of the linearly combined pattern of the first 5 components was also characterized by relative reductions of left temporal and right parietal WMH volumes and relative increases in left and right occipital WMH volume (see Figures 1C,D). After controlling for age and TIV, the pattern significantly predicted right hippocampal volume (*β* = 151.76, *p* = 0.001). The pattern also significantly predicted right hippocampal volume (*β* = 301.15, *p* = 1.13E-8), after we controlled for total WMH volume.

Follow-up univariate correlations revealed that greater right and left hippocampal volumes were significantly related to decreases in left and right frontal, parietal, and temporal WMH volumes (*p’s* < 0.05), but were not related to occipital WMH volumes (see Supplementary Table 1), indicating the relative increases in occipital WMH in the covariance pattern reflect relatively lower decreases or preservation with increasing hippocampal volume.

The expression of the left and right hippocampal-related WMH patterns were highly correlated with each other (*r* = 0.971, *p* = 4.06E-119), indicating a high level of shared variance with similar regional WMH contributions across cerebral hemispheres. A follow-up SSM analysis on the hippocampal volume averaged over both hemispheres showed a very similar pattern as the left and right hippocampal volumes individually. Additionally, SSM analyses, after statistically removing the average hippocampal volume from the left and right hippocampal values, demonstrated that no distinct lateralized patterns were observed (see Supplemental material).

### 3.2 Demographics

Linear regressions including age, sex, education, TIV, APOE ε4 status, and vascular risk score as independent predictors, and left or right hippocampal-related regional WMH patterns as the dependent variables revealed increasing age was significantly associated with lower expression of the left (*β* = −0.048, *p* = 1.80E-13) and right (*β* = −0.036, *p* = 8.38E-11) hippocampal-related WMH patterns. Further, APOE ε4 carriers had only marginally lower expression of the left (*β* = −0.226, *p* = 0.074) and right (*β* = −0.211, *p* = 0.051) hippocampal-related WMH patterns compared to non-carriers. TIV, sex, education, and vascular risk score were not significantly related to either pattern (*p*’s > 0.05; see Supplementary Table 2).

### 3.3 Subjective and objective memory

Linear regressions with age, sex, education, TIV, APOE ε4 status, and vascular risk score as covariates revealed there were no significant relationships between the left hippocampal-related WMH pattern and BSRT sum recall (*β* = −0.627, *p* = 0.685) or CLTR (*β* = −2.06, *p* = 0.479). However, lower expression of the left hippocampal-related WMH pattern was associated with lower MFQ scores (more subjective memory complaints; *β* = 0.234, *p* = 0.032). Similarly, the right hippocampal-related WMH pattern had no significant relationships with BSRT sum recall (*β* = −1.60, *p* = 0.376) or CLTR (*β* = −4.28, *p* = 0.205), but lower expression of this pattern was associated with less MFQ scores (*β* = 0.292, *p* = 0.021).

## 4 Discussion

Using a multivariate covariance approach, our study identified regionally distributed patterns of lobar WMH volume differences associated with left and right hippocampal volume, characterized by relative decreases of WMH volumes in left temporal and right parietal regions, as well as relative preservations of WMH volumes in left and right occipital areas. This suggests hippocampal volume reductions in generally healthy aging may be associated with differences in regional WMH volume distribution. Further, we found lower expression of the left and right hippocampal-related regional WMH patterns were significantly associated with increasing age, and greater subjective memory complaints. In contrast, neither pattern was significantly associated with objective memory performance in this healthy aging cohort, which is similar to previous studies that have observed age-related differences in subjective memory earlier than with measures of objective memory (Dufouil et al., 2005). The findings of the present study indicate that hippocampal-related regional WMH network patterns may be particularly vulnerable to aging and could represent a marker of subjective memory difficulties in healthy older adults.

Few studies have investigated how regional lobar WMH volumes impact hippocampal volume in older adults, and the extant findings are mixed, with separate studies observing associations between hippocampal and WMH volumes within different lobes (Guzman et al., 2013; Rizvi et al., 2018; Vipin et al., 2018; Van Etten et al., 2021). Previous studies have been limited to univariate approaches, whereas multivariate analyses test for regional covariance among brain regions and may be more sensitive for detecting regionally distributed effects in neuroimaging data (Alexander and Moeller, 1994). Our multivariate findings indicate that, within cognitively healthy older adults, *greater* hippocampal volume was associated with decreases in left temporal and right parietal WMH volumes and relative preservations in bilateral occipital WMH volumes. As such, with *smaller* hippocampal volumes, the pattern shows greater WMH volumes in left temporal and right parietal regions and relatively less increases of bilateral occipital WMH volumes. The present findings suggest hippocampal volume reductions may be preferentially sensitive to relative increases of left temporal and right parietal WMH volume in healthy aging. This was consistent across hemispheres, and the left and right hippocampal patterns were highly significantly related. Follow-up analyses with average hippocampal volume also showed a very similar pattern and there were no lateralized results, after we controlled for the average hippocampal volume in the left and right patterns, further supporting highly consistent regional WMH covariance in relation to hippocampal volumes across hemispheres. Notably, previous studies have indicated the posterior cerebral artery (PCA) supplies the hippocampus, temporal, and parietal regions (Brandt et al., 2000; Rizvi et al., 2021). It is possible that reductions in PCA supply may lead to greater accumulation of parietal and temporal WMH volumes, and associated decreases in hippocampal volume within older adults.

In our multivariate approach, we observed occipital WMH volumes were relatively increased with greater hippocampal volumes. Follow-up univariate analyses, however, indicated occipital WMH volumes were not significantly related to left and right hippocampal volumes, whereas all other regional WMH volumes showed significant negative associations with hippocampal volumes. This suggests our multivariate pattern findings may reflect that hippocampal volumes are relatively less associated with elevated WMH volumes in occipital lobes than in other areas. Further, our results may highlight that the relative regional distribution, distinct from the overall total burden, of WMH volume accumulation may be an especially important factor when investigating the effects of regional WMH volume on brain aging.

Although a non-significant trend, we observed APOE ε4 carriers had numerically lower expression of the left and right hippocampal-related regional WMH patterns relative to ε4 non-carriers, suggesting APOE ε4 genotype may only marginally influence hippocampal volume reductions via vascular-related mechanisms in such a healthy aging cohort. Larger samples with greater power to detect smaller effects are needed to further evaluate the role of the APOE ε4 allele in healthy cognitive aging. Additionally, the patterns were not significantly associated with vascular risk, but this could be partially due to our sample consisting of individuals with a low prevalence of vascular-related health conditions. We found age was significantly negatively related to both hippocampal-related regional WMH patterns, which supports that older adults appear to be more susceptible to hippocampal volume reductions related to regional WMH volume accumulation. After we controlled for age, the amount of variance in hippocampal volume explained by the hippocampal-related WMH patterns were reduced, although the network patterns did still display unique effects on left and right hippocampal volumes. This suggests that age may account for a major portion, but not all, of the variance shared between regional WMH and hippocampal volumes, and age-related differences in regional WMH volumes may exacerbate the detrimental effects of brain aging. Although the specific mechanisms of how WMH volume influences gray matter atrophy in aging is unknown, it is possible that increases of WMH volumes in left temporal and right parietal regions with age may influence ischemia-related brain differences, such as hippocampal disconnection and axonal loss via Wallerian degeneration (Schmidt et al., 2011) or tau hyperphosphorylation (Zlokovic, 2011), resulting in hippocampal volume reductions.

Although the hippocampal-related regional WMH volume patterns were not significantly associated with objective memory performance, we found lower expression of our patterns were related to greater subjective memory complaints. A recent study observed that individuals with subjective memory complaints had greater temporal and parietal WMH volume burden, relative to those without complaints (Morrison et al., 2023). Our results expand on these findings and suggest older adults may experience greater subjective memory complaints as a result of differences in regional WMH volumes specifically related to hippocampal volume reductions, which may reflect a potential mechanistic link between the accumulation of temporal and parietal WMH volumes, hippocampal volume reductions, and more subjective memory complaints. Given elevated subjective memory complaints are thought to be a harbinger of future cognitive decline and risk for preclinical AD (Reisberg and Gauthier, 2008), these findings suggest that the hippocampal-related regional WMH network patterns could be a multimodal neuroimaging marker that may be sensitive to very early changes in memory that precede observable differences on objective memory measures in healthy aging. Further research with longitudinal data would be important to test this hypothesis.

In exploratory analyses, we observed significant interactions between vascular risk score and both hippocampal-related WMH patterns on objective, but not subjective memory measures. These results indicated the hippocampal-WMH covariance patterns had greater positive relationships with objective memory performance within the high vascular risk group relative to the low vascular risk group and could suggest that the hippocampal-WMH pattern may be more sensitive to differences in objective memory performance in those with more clinical vascular-related health conditions. As exploratory analyses, however, these findings warrant further investigation to examine the associations in additional cohorts, and especially in those with greater vascular risk burden.

There are several limitations in the current study. The sample was largely White, highly educated, and had relatively low vascular risk burden, which limits the generalizability of our results, and could contribute to the heterogeneity in findings. Further studies with samples that include greater diversity of participants across race, ethnicity, education, and vascular risk are warranted. Additionally, structural and everyday racial discrimination has been associated with reduced hippocampal and WMH volume accumulation (Zahodne et al., 2022), and may exacerbate the impact of regional WMH volume on brain aging (Rizvi et al., 2021). It would be important for future research to investigate how racial discrimination may impact the present findings. Another limitation is our focus on the hippocampus. Although previous findings have shown the hippocampus is particularly sensitive to WMH volume (Fiford et al., 2017), investigating whether and how patterns of regional WMH volume differences are related to other cortical brain volumes would be an important follow up study for future work. Finally, as our data is cross-sectional, more research is needed with longitudinal data to explore and help establish the causal implications of our findings with temporal relationships between regional WMH and hippocampal volumes.

## 5 Conclusion

Within cognitively healthy older adults, we used a multivariate covariance approach to establish multimodal neuroimaging network patterns of regional WMH volume differences related to hippocampal volumes. We identified patterns reflecting increases in left temporal and right parietal WMH volumes and relative decreases of bilateral occipital WMH volumes that were related to smaller left and right hippocampal volumes and were highly associated with age. Although cross-sectional, these findings may potentially reflect disconnection and/or axonal loss through Wallerian degeneration or tau hyperphosphorylation with aging, which may influence hippocampal volume reductions in older adults. Additionally, we observed our hippocampal-related WMH patterns were associated with greater subjective memory complaints. Our findings suggest the identified hippocampal-related WMH patterns may be indicative of early subtle memory difficulties, even before the onset of objective memory deficits. The present study further supports the use of SSM multivariate network analyses with regional WMH volumes to examine the potentially important multimodal associations of regional WMH volumes with brain and cognitive aging in healthy older adults.

## Data availability statement

The raw data supporting the conclusions of this article will be made available by the authors, without undue reservation.

## Ethics statement

This study involving humans was approved by the University of Arizona IRB committee. The study was conducted in accordance with the local legislation and institutional requirements. The participants provided their written informed consent to participate in this study.

## Author contributions

EVE: Conceptualization, Formal analysis, Visualization, Writing – original draft. PB: Formal analysis, Visualization, Writing – review & editing. MG: Data curation, Writing – review & editing. DR: Data curation, Writing – review & editing. GH: Data curation, Writing – review & editing. MH: Data curation, Writing – review & editing. TT: Data curation, Writing – review & editing. GA: Data curation, Formal analysis, Funding acquisition, Project administration, Writing – review & editing.

## Footnotes

^1^ www.itksnap.org
