# Supplementary material for: Regional covariance of white matter hyperintensity volume patterns associated with hippocampal volume in healthy aging
Source: Front Aging Neurosci. 2024 Mar 8;16:1349449. doi: 10.3389/fnagi.2024.1349449 (PMC10957632; doi:10.3389/fnagi.2024.1349449)

Supplementary Material

Regional covariance of white matter hyperintensity volume patterns associated with hippocampal volume in healthy aging

**Emily J. Van Etten^1,2^, Pradyumna K. Bharadwaj^1,2^, Matthew D. Grilli^1,2,3^, David A. Raichlen^4,5^, Georg A. Hishaw^3^, Matthew J. Huentelman^2,6,7^, Theodore P. Trouard^2,7,8^, &**

**Gene E. Alexander*^1,2,7,9,10,11^**

^1^Department of Psychology, University of Arizona, Tucson, AZ, USA

^2^Evelyn F. McKnight Brain Institute, University of Arizona, Tucson, AZ, USA

^3^Department of Neurology, University of Arizona, Tucson, AZ, USA

^4^Human and Evolutionary Biology Section, Department of Biological Sciences, University of Southern California, Los Angeles, CA, USA.

5Department of Anthropology, University of Southern California, Los Angeles, CA, USA.

^6^Neurogenomics Division, The Translational Genomics Research Institute (TGen), Phoenix, AZ, USA

^7^Arizona Alzheimer’s Consortium, Phoenix, AZ, USA

^8^Department of Biomedical Engineering, University of Arizona, Tucson, AZ, USA

^9^Department of Psychiatry, University of Arizona, Tucson, AZ 85721, USA

^10^Neuroscience Graduate Interdisciplinary Program, University of Arizona, Tucson, AZ 85721, USA

^11^Physiological Sciences Graduate Interdisciplinary Program, University of Arizona, Tucson, AZ 85721, USA

*** Correspondence:** Gene E. Alexander, PhD: gene.alexander@arizona.edu

**Supplementary Table 1.** Univariate Pearson correlations between left and right hemispheres of the regional lobar WMH volumes and left and right hippocampal volumes.

| Hippocampal Volume | Regional WMH volume | *r* | *p* | |
| --- | --- | --- | --- | --- |
| Right hippocampus | **Left frontal WMH volume** | **-.256** | | **3.2E-4** |
|  | **Right frontal WMH volume** | **-.318** | | **6.0E-6** |
|  | **Left parietal WMH volume** | **-.243** | | **.001** |
|  | **Right parietal WMH volume** | **-.366** | | **1.6E-10** |
|  | **Left temporal WMH volume** | **-.340** | | **1.0E-6** |
|  | **Right temporal WMH volume** | **-.230** | | **.001** |
|  | Left occipital WMH volume | -.029 | | .687 |
|  | Right occipital WMH volume | -.098 | | .172 |
| Left hippocampus | **Left frontal WMH volume** | **-.321** | | **5.0E-6** |
|  | **Right frontal WMH volume** | **-.355** | | **3.8E-10** |
|  | **Left parietal WMH volume** | **-.280** | | **7.8E-5** |
|  | **Right parietal WMH volume** | **-.366** | | **1.5E-10** |
|  | **Left temporal WMH volume** | **-.357** | | **3.3E-10** |
|  | **Right temporal WMH volume** | **-.252** | | **4.0E-4** |
|  | Left occipital WMH volume | -.062 | | .389 |
|  | Right occipital WMH volume | -.113 | | .118 |

Bolded effects are significant. Abbreviations: WMH = white matter hyperintensity.

**Supplementary Table 2.** Linear regression effects of Age, TIV, sex, education, vascular risk, and APOE ε4 status on the left and right hippocampal-related WMH volume patterns.

| Hippocampal-related WMH volume pattern | Factor | *β* | *p* |
| --- | --- | --- | --- |
|  | Age | -.036 | 8.38E-11 |
| Right hippocampal-related WMH volume pattern | TIV | .290 | .536 |
|  | Sex | -.021 | .870 |
|  | Education | .016 | .417 |
|  | Vascular Risk | -.046 | .284 |
|  | APOE ε4 Status | -.211 | .051 |
|  | Age | -.048 | 1.80E-13 |
| Left hippocampal-related WMH volume pattern | TIV | .020 | .970 |
|  | Sex | .083 | .582 |
|  | Education | .008 | .733 |
|  | Vascular Risk | -.068 | .169 |
|  | APOE ε4 Status | -.226 | .074 |

Abbreviations: APOE = Apolipoprotein, WMH = white matter hyperintensity, TIV = total intracranial volume.

**Supplementary Exploratory Analyses:**

SSM:

We conducted a SSM analysis on hippocampal volume averaged across cerebral hemipheres using the same procedures outlined in the main manuscript. The average hippocampal-related regional WMH pattern model included the first 5 components and accounted for 20.20% of the variance in average hippocampal volume (*β*=298.93, *p*=5.29E-10) with higher expression of the network pattern related to greater volume. Bootstrap re-sampling of the linearly combined pattern of the first 5 components was characterized by reductions of left temporal and right parietal WMH volumes and relative increases in left and right occipital WMH volumes. This pattern was similar to and included the same regions as the left and right hippocampal-related WMH patterns described in the main text. After controlling for age and TIV, the pattern significantly predicted average hippocampal volume (*β*=105.08, *p*=.005). The pattern also significantly predicted average hippocampal volume (*β*=253.86, *p*=3.10E-8), after we controlled for total WMH volume. Additionally, follow-up SSM analyses, after we statistically removed the average hippocampal volume from the left and right hippocampal values, revealed no distinct lateralized patterns.

Linear Regressions:

Linear regressions were conducted to examine interactive relationships between important demographic and clinical characteristics (including age, sex, education, APOE ε4 status, and vascular risk) and the left or right hippocampal-related regional WMH patterns with objective memory performance and subjective memory complaints as the dependent variables. All linear regressions included the other demographic characteristics, as well as total intracranial volume (TIV) as covariates.

There were significant interactions of vascular risk by left hippocampal-WMH pattern on both objective memory measures (sum recall and CLTR), but not subjective memory complaints. Additionally, there were significant interactions of vascular risk by right hippocampal-WMH pattern on both objective memory measures (sum recall and CLTR), but not subjective memory complaints (see Supplementary Table 3).

All significant interactions showed the same pattern of results with the hippocampal-WMH covariance patterns having greater positive relationships with objective memory performance within the high vascular risk group (those with 2 or more vascular risk factors) relative to the low vascular risk group (individuals with 0 or 1 vascular risk factors). These associations are depicted in Supplementary Figures 1 and 2, which show relationships between left (see supplementary Figure 1) and right (see supplementary Figure 2) hippocampal-related WMH volume patterns and SRT CLTR performance between the low and high vascular risk groups.

There were no significant interactions between the left or right hippocampal-WMH volume pattern and any other demographic or clinical characteristic on subjective or objective memory measures (see Supplementary Table 3).

**Supplementary Table 3.** Interactive effects of age, sex, education, vascular risk, and APOE ε4 status with left and right hippocampal-related WMH volume patterns on subjective and objective memory measures.

| Interaction variables | Dependent Variable | *β* | *p* |
| --- | --- | --- | --- |
| Age by left hippocampal-related WMH volume pattern | Subjective memory complaints | .141 | .495 |
|  | SRT Sum Recall | 2.88 | .371 |
|  | SRT CLTR | 6.99 | .224 |
| Age by right hippocampal-related WMH volume pattern | Subjective memory complaints | .148 | .550 |
|  | SRT Sum Recall | 1.04 | .787 |
|  | SRT CLTR | 5.77 | .422 |
| Sex by left hippocampal-related WMH volume pattern | Subjective memory complaints | -.032 | .857 |
|  | SRT Sum Recall | 3.41 | .183 |
|  | SRT CLTR | 1.48 | .759 |
| Sex by right hippocampal-related WMH volume pattern | Subjective memory complaints | .016 | .941 |
|  | SRT Sum Recall | 2.99 | .345 |
|  | SRT CLTR | .481 | .935 |
| Education by left hippocampal-related WMH volume pattern | Subjective memory complaints | .014 | .713 |
|  | SRT Sum Recall | -.156 | .774 |
|  | SRT CLTR | -.625 | .539 |
| Education by right hippocampal-related WMH volume pattern | Subjective memory complaints | .000 | .996 |
|  | SRT Sum Recall | .180 | .787 |
|  | SRT CLTR | -.314 | .801 |
| APOE ε4 status by left hippocampal-related WMH volume pattern | Subjective memory complaints | -.113 | .603 |
|  | SRT Sum Recall | 2.78 | .371 |
|  | SRT CLTR | 2.76 | .636 |
| APOE ε4 status by right hippocampal-related WMH volume pattern | Subjective memory complaints | -.189 | .479 |
|  | SRT Sum Recall | 2.23 | .552 |
|  | SRT CLTR | 2.75 | .703 |
| Vascular risk by left hippocampal-related WMH volume pattern | Subjective memory complaints | .050 | .579 |
|  | **SRT Sum Recall** | **3.57** | **.006** |
|  | **SRT CLTR** | **6.59** | **.007** |
| Vascular risk by right hippocampal-related WMH volume pattern | Subjective memory complaints | .047 | .679 |
|  | **SRT Sum Recall** | **4.48** | **.006** |
|  | **SRT CLTR** | **8.22** | **.007** |

Abbreviations: WMH = white matter hyperintensity, SRT = Selective Reminding Test, CLTR = consistent long-term retrieval

**Supplemental Figure 1.** Relationship between left hippocampal-related WMH volume pattern and SRT CLTR performance between the low and high vascular risk groups.


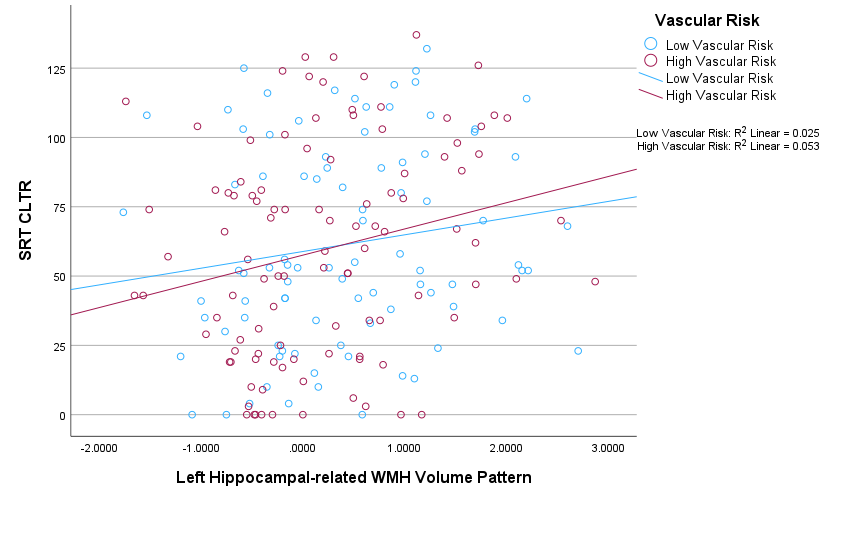


**Supplemental Figure 2.** Relationship between right hippocampal-related WMH volume pattern and SRT CLTR performance between the low and high vascular risk groups.


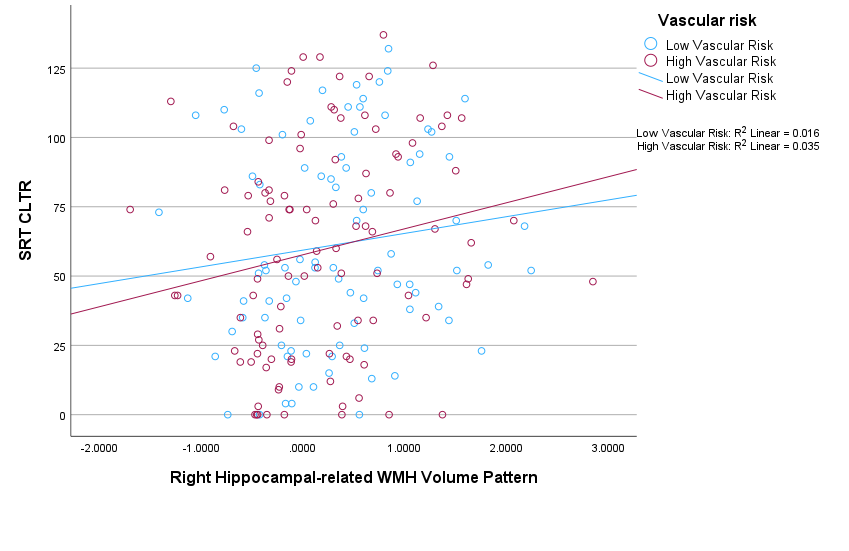

Supplement: Supplementary file 1 [file Data_Sheet_1.docx]
